# Supplementary material for: Splice-Junction-Based Mapping of Alternative Isoforms in the Human Proteome
Source: Cell Rep. Author manuscript; Available in PMC 2020 Jan 15. (PMC6961840; doi:10.1016/j.celrep.2019.11.026)
Supplement: 2 [file NIHMS1546469-supplement-2.zip › DF1/PXD006675/LeftAtrium/LeftAtrium_18_TNS1_PHNPADILLHPTGEEDEGK.pdf]

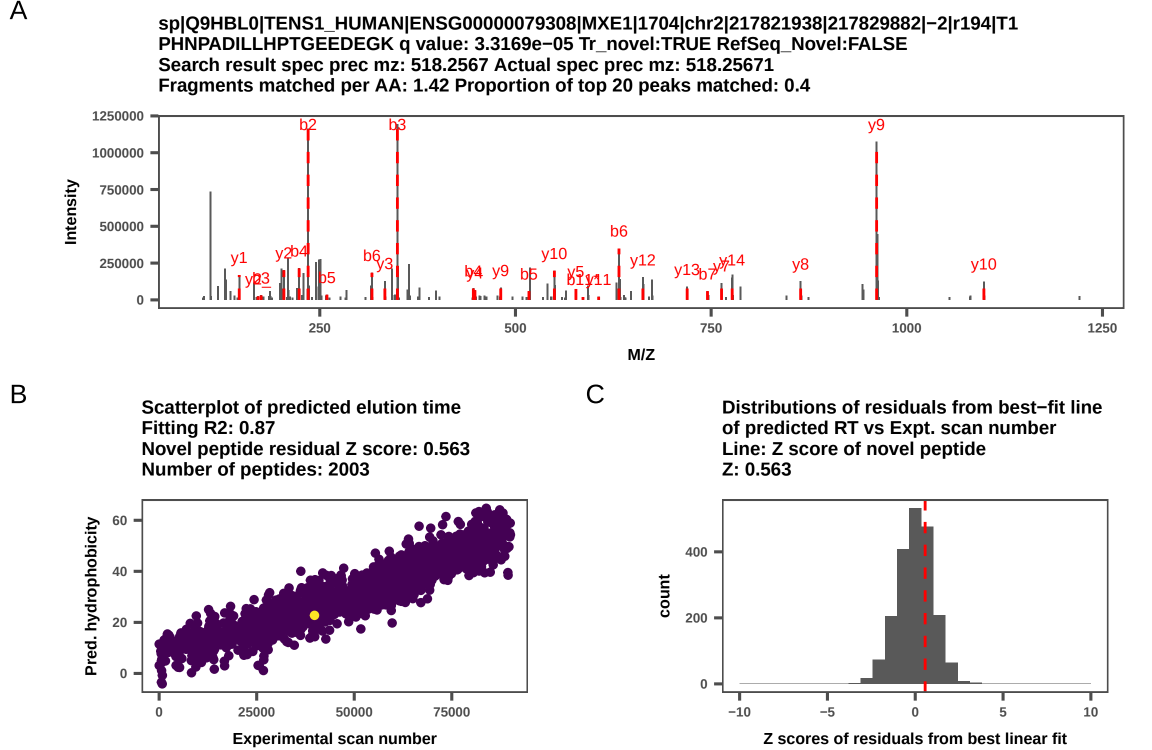
A
sp|Q9HBL0|TENS1_HUMAN|ENSG00000079308|MXE1|1704|chr2|217821938|217829882|−2|r194|T1
PHNPADILLHPTGEEDEGK q value: 3.3169e−05 Tr_novel:TRUE RefSeq_Novel:FALSE
Search result spec prec mz: 518.2567 Actual spec prec mz: 518.25671
Fragments matched per AA: 1.42 Proportion of top 20 peaks matched: 0.4
Intensity
M/Z
b2
b3
y9
b6
y10
y12
y14
y8
y1
b5
y13
b7
y7
y5
y11
0
250000
500000
750000
1000000
1250000
250
500
750
1000
1250
B
Scatterplot of predicted elution time
Fitting R2: 0.87
Novel peptide residual Z score: 0.563
Number of peptides: 2003
Pred. hydrophobicity
Experimental scan number
0
20
40
60
0
25000
50000
75000
C
Distributions of residuals from best−fit line
of predicted RT vs Expt. scan number
Line: Z score of novel peptide
Z: 0.563
count
Z scores of residuals from best linear fit
0
200
400
−10
−5
0
5
10
